# Supplementary material for: Patterns of Intron Gain and Loss in Fungi
Source: PLoS Biol. 2004 Nov 30;2(12):e422. doi: 10.1371/journal.pbio.0020422 (PMC532390; doi:10.1371/journal.pbio.0020422)
Supplement: Table S1 — Also available at http://genes.mit.edu/NielsenEtAl/. (4.3 MB ZIP). [file pbio.0020422.st001.zip › NielsenEtAl/html/51.html]

AN3826.1.NCU00924.1.MG01168.1.FG00694.1


```
 CLUSTAL W (1.82) Multiple Sequence Alignments - Introns Inserted


Sequence 1: NCU00924.1	427 aa
Sequence 2: MG01168.1	451 aa
Sequence 3: FG00694.1	421 aa
Sequence 4: AN3826.1	407 aa
Alignment Length: 451 aa
Number Identitical Residues: 197 aa
Alignment Score (without introns) 10383


MG01168.1 	MNAGTARTTSRPSGRPLGIYIAGAALRLALFTLFPGLPDLLTGRVEISTPVTSFKRL1TF
NCU00924.1	MNGG--KVTLR---RTAGLYAGAAVLRLALFTLFPGLPDLLTGRVEISTPVTSFKRL1--
FG00694.1 	-------MTLR---GKASVFAGAALLRLVLFLAFPGLPNLLTGRVEISTPVTSFKRL1--
AN3826.1  	------MPVDR---RKVAVFGGALALRLLLLLLFPSLPDLLTGRVEVSTPVTSFKRL1--
          	        . *      .:: ..  *** *:  **.**:*******:**********   

MG01168.1 	SSTDTLSSLPAPTVQEGLFLYNHNVSPYDGGVYHQ~APLLLPLFSLLPDVATYPIFTALL
NCU00924.1	--------------QEGLFLYNHNVSPYDGGVYHQ~APLLLPIFNLLPSFAAFPIFTYLL
FG00694.1 	--------------QEGLFLYTNNVSPYEGGVFHQ~APLLLPLFSLLPDVKSWPIFTHLL
AN3826.1  	--------------QEGLFLYNRNVSPYDGGVFHQ0----------------YPIPTALL
          	              *******..*****:***:**                 :** * **

MG01168.1 	YIAVDLFCADALLQIAESGEAGSSAKFQSPRRQRRWSGAIVAAI2YLFSPFTIATCIGRS
NCU00924.1	YIAIDILSAHALLRIAESGEASSSARFTSPRRDKRWSGAIIAAL~FLFNPFTIATCIGRS
FG00694.1 	YIAVDLLSADALYKIADSGVASNSRLFKSPRRANKFGTAAVAAG2FLFNPYTIATCIGRS
AN3826.1  	YSLVDLLNANALVTISDSAQAVSGRLYTSSRKLIKWDGIAVAAW2FLFNPFTIATCLGRS
          	*  :*:: *.**  *::*. * ..  : *.*:  ::.   :**  :**.*:*****:***

MG01168.1 	TAVFTSCAVLHAIAKAIQGRTLGALVSIAFASYLSMSPLLLLPPLLVLAYDRQPPAPARA
NCU00924.1	TTVFTTCAILHAVANAISGRPFHAMVALAFASYLSMYPLLLLPPLILLCYDRQK-LPATG
FG00694.1 	TGVFTNCAILLAITKAIQGSPFNAMVALSFASYLSMYPILLLPPLVLLAYDCQV--EKRR
AN3826.1  	TAVFTSTGILYAISAAVQGESLNAMFALGLASYLSIYPALLFIPLILLCYDRHA---QRS
          	* ***. .:* *:: *:.* .: *:.::.:*****: * **: **::*.** :       

MG01168.1 	HLSASPAKFAVVGTGVVMAVLAVLAALSLVITAFSWEFLASTYGIQLTLADLTPNAGLWW
NCU00924.1	APNAW-IKFTATMVMDAFGALAVLFQMSFFLTG-SWEFLSSTYGVQLTLSDLAPNIGLWW
FG00694.1 	VTSSS--KFATTNVAVVAGCMVSLLGMSFLLANNSWEFLARTYGIQLTLSDLTPNVGLWW
AN3826.1  	QSSPSTPLFVAKHLAILLASIAGLLGISVLIIGDFSNLISATYGFQLLVPDLTPNIGLWW
          	  .. .  *..       . :. *  :*..:     :::: ***.** :.**:** ****

MG01168.1 	YFFIEMFDPFRSFFLAVFWLHLASYVGGLTVRIRTQPLAALSILLGLFAIFKPYPSIADT
NCU00924.1	YFFVEMFDSFRSFFLAVFWLHLSSYVGGLSVRLRQQPLAVITLLLGIFSIFKPYPSIADA
FG00694.1 	YFFIEMFDSFRAFFLGVFWLHLASYPAALSIRLRPQPLAVLTILLGTFSIFKPYPSLADA
AN3826.1  	YFFIEIFDSFRDFFLGVFWLHLAAYAGSLSVRLRRQPLFVVTTLLGIFAVFKPYPSISDA
          	***:*:**.** ***.******::* ..*::*:* *** .:: *** *::******::*:

MG01168.1 	ALFLGLLPLFRHVFPL~MRYPFVIAAVIVYAVFLGPAFWHLWIYAGSGNANFFYAITLVW
NCU00924.1	SLFLGMVPLYRHVFPL~LRYSFVIAAIIIYTTFLGPAFYHLWIYAGSGNANFFYAITLVW
FG00694.1 	SLFLSVVPLFRHVFPL~MRYAFVTTSTLLYATFLGPAFYHLWIYAGSGNANFFYAITLVW
AN3826.1  	SLYFAVLPIYRHLFPL1MRYTFFSVSALLYASLLGPAFYHLWIYAGSGNANFFYAITLVW
          	:*::.::*::**:*** :**.*. .: ::*: :*****:*********************

MG01168.1 	SLVQSLLVSDFTFALLRDEWEVDRPEMVGKEIRQI
NCU00924.1	GLGLSLLVCDLAFAVLRDEWEVERPEMAGKEIRQI
FG00694.1 	SLGQSLLVTDLTFAVLRDEWEIDRPEMAGKEIRQI
AN3826.1  	SLGFSLILADMIFAALRDEWEQENPDKRGKPVKQV
          	.*  **:: *: ** ****** :.*:  ** ::*:
```
